# Signalling in systemic plant defence – roots put in hard graft

**DOI:** 10.1093/jxb/erw349

**Published:** 2016-10-13

**Authors:** Simon C. Groen

**Affiliations:** Department of Biology and Center for Genomics and Systems Biology, New York University, New York, NY 10003, USA

**Keywords:** Citrus, glutamate, herbivore, induced systemic resistance, jasmonic acid, oxylipin, pathogen, plant defence, systemic acquired resistance, systemic signalling, two-spotted spider mite (*Tetranychus urticae*).

**Roots are increasingly recognized as key regulators of aboveground interactions between plants and other organisms. In this issue of *Journal of Experimental Botany*, Agut *et al.* (pages 5711–5723) enrich our understanding of the underground signalling mechanisms in the shoot–root–shoot (SRS) loop that regulates canopy-wide defence responses after a leaf is attacked.**

When it comes to keeping the world green, roots have historically been considered to be mere purveyors of water and nutrients to the shoots. However, this view has changed dramatically over the past 25 years as evidence has accumulated that roots can be the ‘movers and shakers’ in orchestrating aboveground interactions between plants and their panoply of parasites (Bezemer and van Dam, 2005; Erb *et al.*, 2009; Pieterse *et al.*, 2014).

Through the use of ingenious combinations of functional genetics and micro-grafting, signalling mechanisms involving the root system have been identified that alter the level of resistance to aboveground attacks (Rudrappa *et al.*, 2008; Erb *et al.*, 2009; Nalam *et al.*, 2012; Fragoso *et al.*, 2014). Levels of shoot resistance can also be influenced by beneficial and harmful interactions between roots and a variety of soil-inhabiting organisms (Bezemer and van Dam, 2005; Pieterse *et al.*, 2014). In addition to regulating aboveground defences, roots serve as dynamic producers and storage facilities for defensive metabolites and nutrients that can be deployed aboveground through vascular transport (Erb *et al.*, 2009).

The importance of roots in defence against aboveground attackers has unfortunately become painfully obvious to citrus growers, who have seen their orchards become infested by a trinity of leaf-feeding herbivores with piercing–sucking lifestyles. In the Americas, leaf attacks by the Asian citrus psyllid *Diaphorina citri* have caused an epidemic of citrus greening disease (also known as Huanglongbing). The *Candidatus Liberibacter* spp. bacteria spread by the psyllid ravaged millions of commercial citrus trees. Combinations of rootstocks and scions have now been identified that show tolerance to high Huanglongbing pressure (Stover *et al.*, 2016). These promising results will hopefully contribute to a successful disease management programme.

On both sides of the Atlantic, the brown citrus aphid *Toxoptera citricida* has long spread citrus tristeza virus (CTV) between leaves, which has sent more than 85 million trees to an untimely retirement (Bruessow *et al.*, 2010). Although CTV-tolerant rootstocks have been identified and deployed in the groves, this is likely to have contributed to outbreaks of the third herbivore, the two-spotted spider mite *Tetranychus urticae*. Heavy spider mite infestations lead to fruit scarring, chlorotic leaf spots and leaf loss (Bruessow *et al.*, 2010).

## A holistic view of systemic signalling in plant defence

With these problems in mind, Agut and colleagues took the results of a seminal study by Karban and Carey (1984), who identified that leaf infestation by spider mites can induce systemic resistance to secondary attacks, and applied these to understanding defence regulation in citrus trees using a more holistic approach. Where previous studies of shoot-induced systemic resistance often focused solely on between-leaf signals (Fu and Dong, 2013; Mousavi *et al.*, 2013), Agut *et al.* followed the few examples in which roots were explicitly considered as taking part in the regulation of leaf-initiated aboveground defences (e.g. Rudrappa *et al.*, 2008; Erb *et al.*, 2009; Nalam *et al.*, 2012; Fragoso *et al.*, 2014).

The authors previously identified two citrus rootstocks, sour orange (*Citrus aurantium*) and Cleopatra mandarin (*C. reshni*), with different levels of susceptibility to spider mites. Sour orange leaves show fewer symptoms than Cleopatra mandarin leaves, and spider mites prefer and perform better on the latter. The higher level of resistance in sour orange is caused in part by a stronger induction of oxylipin signalling, key in regulating anti-herbivore defences in many plant species (Agut *et al.*, 2014). Furthermore, spider mite feeding induces the production of leaf volatile chemicals that repel conspecifics on sour orange, while the exact opposite effect is observed in Cleopatra mandarin (Agut *et al.*, 2015).

In the current study, Agut *et al.* grafted clementine (*C. clementina*) scions onto Cleopatra mandarin and sour orange rootstocks, and observed that spider mite-induced systemic resistance to secondary attacks was graft-transmissible. Although present in scions grafted onto either rootstock, the systemic resistance was stronger in scions attached to sour orange rootstocks. Metabolite profiling of sour orange and Cleopatra mandarin scions and rootstocks showed that the mobile signals responsible for the systemic resistance surprisingly differed between genotypes. In Cleopatra mandarin, spider mite-induced systemic resistance correlated with enhanced leaf efflux of myo-inositol and elevated abscisic acid (ABA) levels in systemic leaves. In sour orange, spider mite feeding induced the transport of Glu, 2-hydroxyglutarate, citric acid and two fatty acids to systemic leaves and the roots. In turn, sour orange roots also increased the export of Glu to the scion, to which the systemic leaves responded by increasing the expression levels of *GLUTAMATE RECEPTOR-LIKE* (*GRL*) genes. GRL protein-mediated signalling activates oxylipin signalling (Mousavi *et al.*, 2013), and levels of the oxylipins jasmonic acid and 12-oxophytodienoic acid were elevated in systemic leaves (Agut *et al.*, 2016). Since oxylipin signalling is necessary for resistance to spider mites (Agut *et al.*, 2014), Agut and colleagues have now come full circle in laying out the basic framework for the regulation of spider mite-induced systemic resistance to secondary attacks.

## The shoot–root–shoot (SRS) loop in plant defence

The findings contribute to a growing body of evidence for the existence of an integrated SRS loop that regulates systemic defences after detection of an initial attack (Box 1). SRS loops have now been observed to regulate defence against a variety of pests and pathogens – including bacteria (Rudrappa *et al.*, 2008), and herbivores with chewing (Erb *et al.*, 2009; Fragoso *et al.*, 2014) and piercing–sucking feeding habits (Nalam *et al.*, 2012; Fragoso *et al.*, 2014; Agut *et al.*, 2016; Kim *et al.*, 2016) – across the angiosperms: monocots (maize), rosid eudicots (Arabidopsis, citrus) and asterid eudicots (wild tobacco, pepper).

**Box 1. The shoot–root–shoot (SRS) loop in plant defence**

When an attack on the leaves by herbivores or pathogens (yellow lightning strike) is recognized by the plant, a cascade of signalling events is set in motion. From the site of attack systemic signals are sent out to other leaves and the roots. These signals include, but are not limited to, Glu, citric acid, fatty acids and myo-inositol (Agut *et al.*, 2016). The intricacies of these signals have been reviewed elsewhere (Fu and Dong, 2013; Mousavi *et al.*, 2013). Roots may respond to these signals by releasing oxylipins (Nalam *et al.*, 2012) or additional Glu (Agut *et al.*, 2016), producing defensive metabolites (Erb *et al.*, 2009; Fragoso *et al.*, 2014), or recruiting beneficial microbes (Rudrappa *et al.*, 2008; Pieterse *et al.*, 2014; Kim *et al.*, 2016). Root-derived defensive metabolites (Erb *et al.*, 2009; Fragoso *et al.*, 2014) and/or signals are then sent back aboveground where they contribute to the regulation of canopy-wide defences. The systemic defence response to herbivores relies on active signalling by abscisic acid and oxylipins such as jasmonic acid and 12-phytodienoic acid (Erb *et al.*, 2009; Nalam *et al.*, 2012; Fragoso *et al.*, 2014; Pieterse *et al.*, 2014; Agut *et al.*, 2016). The colours of the arrows correspond to the plant organs involved: leaves, petioles and branches (green); stem (brown); and roots (beige).

**Figure:**
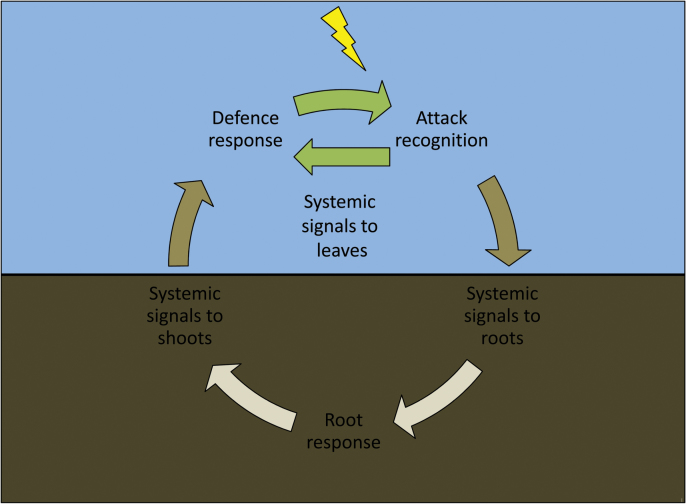

Although all of these studies point to the existence of SRS loops in plant defence, there is currently no full overlap between them in the mechanistic details that have been described. However, some overarching themes seem to emerge. One is the requirement of intact oxylipin signalling in both above- and belowground tissues (Erb *et al.*, 2009; Nalam *et al.*, 2012; Agut *et al.*, 2014, 2015, 2016; Fragoso *et al.*, 2014). A second is the involvement of Glu metabolism and perhaps the tricarboxylic acid (TCA) cycle (Seifi *et al.*, 2013). Glu can activate oxylipin signalling (Mousavi *et al.*, 2013), and the findings of Agut *et al.* (2016) are fully in line with that. The TCA cycle generates energy that fuels metabolic reactions during plant defence responses (Seifi *et al.*, 2013). The up-regulation of metabolites involved in the TCA cycle – such as Glu, 2-hydroxyglutarate, citric acid and malic acid – point to a role for the TCA cycle in SRS loops (Rudrappa *et al.*, 2008; Agut *et al.*, 2016). Lastly, ABA seems to be an important signal in at least a subset of interactions (Erb *et al.*, 2009; Fragoso *et al.*, 2014; Agut *et al.*, 2016).

The elegant series of studies by Agut *et al.* (2014, 2015, 2016) have done much to ‘close the SRS loop’ and pave the way for future functional studies that will further enrich our understanding of the plant defence system. With the genome sequences for clementine and sour orange available (Wu *et al.*, 2014) one could start to explore the genetic basis of the systemic defence regulatory mechanisms. Profiling levels of mRNAs and small RNAs, which can also cross the above- and belowground boundary to direct gene expression in distal organs (Lewsey *et al.*, 2016), will lead to a more-detailed understanding of systemic defence signalling, especially when done in conjunction with metabolite profiling.

The work by Agut *et al.* (2014, 2015, 2016) and other studies on systemic defence signalling (reviewed in Erb *et al.*, 2009; Fu and Dong, 2013; Pieterse *et al.*, 2014) have so far identified a multitude of long-distance signals that regulate plant defence. Why are there so many? Does redundancy between signals provide robustness in the face of subversive manipulation by attackers, or do different combinations of signals confer specificity (Kim *et al.*, 2014)? Whatever the answer, root signals will be at the heart of it.
